# Report of Nine Cases of Gun-Shot Wound of the Elbow Joint, with Partial or Total Excision in Seven Cases

**Published:** 1866-07

**Authors:** J. J. Knott

**Affiliations:** Surgeon late Confederate army


### CORRESPONDENCE.

---

### ARTICLE IV.

*Report of nine cases of gun-shot wound of the elbow joint, with partial or total excision in seven cases.* By J. J. KNOTT, M. D., Surgeon late Confederate army.

Such has been the success attending excision of the elbow joint, that it has became, of late years, an operation of very frequent performance. Though we are sorry to say that during the past war, this operation was often discarded either through the ignorance or prejudice of the surgeons, and that of amputation substituted, being the means not only of increasing the lists of mortality, but depriving the men of useful limbs.

Experience has conclusively proved to us, that the dangers consequent upon excision of this joint, in the majority of instances, are very slight. A great many army surgeons, I am aware, raised serious objections to excision on account of the distance it was often necessary to transport the patients in ambulances. According to our experience and observation, the patients who had submitted to excision, bore transportation much better, in every respect, than those who had undergone amputation. It is concluded by some surgeons that where the medullary canal of the humerus is involved in the exsection, that the dangers of diffuse suppuration and pyemia are increased. While we willingly admit that this may be the case in caries and other diseases of the osseous system, we must emphatically deny that such is the case in operations following gun-shot wounds. Again, great stress is laid upon saving as much bone as possible. Authors contend that the use of the limb will be increased in proportion to the amount of bone spared. Here, again, is another point calculated to lead the inexperienced into error. We never have, except in one instance, (case No. 7) been satisfied in removing less than the whole articular surface of the joint; and we will here state, that in those cases where we removed the condyles entire, we had the most favorable recoveries. So, while the operator should be careful and not remove too much bone, he should be equally impressed with the important fact of not leaving too much. As regards the manner in which the incision should be made, we find a diversity of opinion existing amongst writers upon this subject. Morean, the first to put this operation into practice, used an H incision; while Park, the one that first suggested it, employed a simple longitudinal incision; while by others, different modes of procedure have been adopted. In our judgment, where the operation is called for in consequence of disease of the joint, the operation should be modified by the operator to meet the exigencies of the case. Now in gun-shot wounds, of which we write more particularly, we have followed the simple procedure of Park, i. e., the longitudinal incision. The following is the manner in which we have generally proceeded in this operation:

The patient being placed upon his back on the operating table, is completely anæthetised, after which he is placed upon his side opposite to injured limb, and retained in this position by an assistant. The arm is then brought over the edge of the table and held in a semi-flexed position by an assistant; I then commence my incision one half inch below the line of articulation, between the bones of the forearm and humerus, extending my incision upwards, on a line, and directly over the olecranon process of the ulnar, the distance of four inches above the commencement cut of the incision; the last half inch extending through the integument only. After carefully dissecting the ulnar nerve out of the groove on the internal surface of the olecranon process and its trochlea on internal condyle, and putting it aside by means of a blunt hook or the handle of an ordinary scalpel, we divide the tendon of the *triceps extensor cubiti* and the ligamentons attachments of the point. Now by forcibly flexing the arm, we are enabled to divide the attachments around the bones of forearm sufficiently to remove their articular surfaces, which should be done with the saw (either chain or amputating) and not with the pliers, as recommended by authors, as the bones are very often crushed through instead of cut by this means. We next proceed to the removal of the condyles, which is done by means of the saw, after they have been sufficiently cleared of their attachments. Care must be taken in our dissections not to wound the anterior brachial muscles, as they are still to fulfill their important functions, as well as danger of wounding the brachial artery. After clearing the parts of all extraneous matter, the lips of the incision are approximated by means of the interrupted sutures, with intermediate strips of adhesive plaster; cold water dressings applied, and the forearm retained in a position at right angles with the humerus by means of two angular splints, applied latterly and retained by a roller bandage extending from hand to upper part of splint, we need have little fear of anchylosis, provided passive motion is properly employed.

Case No. 1.—Corporal P——, Company "E," 53d Georgia Regiment, Semmes' Brigade, McLaws' Division, Longstreet's Corps, Army Northern Virginia; aged about thirty; dark hair; dark complexion; and by occupation, previous to enlistment, a farmer. Received in the battle of Chancellorsville, Virginia, a gun-shot wound through right elbow; the ball passing transversely through the joint, fracturing both condyles and olecranon process of ulnar.

*Operation.*—Removed both condyles with the olecranon process and head of radius.

*Result.*—Patient recovered with useful limb.

Case No. 2.—First Sergeant, Company —— Regiment Pennsylvania Volunteers; aged about twenty-five years; light hair; fair complexion; and by occupation, previous to enlistment, apothecary. Received at the battle of Chancellorsville, Virginia, a gun-shot wound through left elbow, fracturing both condyles.

*Operation.*—Removed both condyles, olecranon process and head of radius.

*Result.*—I saw this patient seven days after the operation, at which time he was doing finely, and I have no doubt recovered with a useful limb.

Case No. 3.—Sergeant A——, Company "A," 53d Georgia Regiment, Semmes' Brigade, McLaws' Division, Longstreet's Corps, Army Northern Virginia; hair light; complexion fair; and by occupation, previous to enlistment, a farmer. Received in the battle of Gettysburg, Pennsylvania, a gun-shot wound through right elbow, fracturing both condyles, olecranon process and head of radius.

*Operation.*—Removed both condyles, olecranon process and head of radius.

*Result.*—Patient recovered with useful limb, and afterwards died, while a prisoner, with "typhoid dysentery."

Case No. 4.—Private J——, Company "B," 53d Georgia Regiment, Bryant's Brigade, McLaws' Division, Longstreet's Corps; aged eighteen years; health bad; complexion sallow; hair dark; and by occupation, previous to enlistment, a farmer. Received in the assault on Fort Sanders, Knoxville, Tennessee, a gun-shot wound (flesh) through right arm, some three inches below the shoulder. Not leaving his command for this, he received a second through the left elbow—the ball entering some two inches below the articulation of the elbow on the radial and outer side, and passed obliquely through the joint, producing a fracture of radius and both condyles.

*Operation.*—Removed three inches of the radius and both condyles, with olecranon process of the ulnar.

*Result.*—Patient recovered with a useful limb.

Case No. 5.—Private H——, Company "C," 53d Georgia Regiment, Semmes' Brigade, Kershaw's Division, Longstreet's Corps; aged seventeen years; health good; hair dark; complexion fair; and by occupation, previous to enlistment, a farmer. Received at the battle of Berryville, Shenandoah Valley, Virginia, a gun-shot wound through the right elbow, fracturing both condyles and olecranon process of ulnar.

*Operation.*—Removed both condyles and olecranon process of ulnar.

*Result.*—Recovered with useful limb.

Case No. 6.—Lieutenant B——, color bearer, 51st Georgia Regiment, Semmes' Brigade, Kershaw's Division, Longstreet's Corps. Received during the battle of Cedar Creek, Valley of Virginia, a gun-shot wound through the left arm, fracturing both condyles.

*Operation.*—By request of my friend, Surgeon E. M. Watt, I operated on this case. I removed the condyles and olecranon process.

*Result.*—Recovered with useful limb.

CASE No. 7.—Sergeant P——, Company "B," 53d Georgia Regiment, Kershaw's Division, Longstreet's Corps, Army Northern Virginia; hair dark; complexion fair. Received at the battle of Cold Harbor, June 1st, 1864, a gun-shot wound in right elbow, fracturing olecranon process, and laying the joint open.

*Operation.*—Removed olecranon process, and approximated edges of wound by means of the interrupted suture.

*Result.*—Recovered with complete anchylosis of the joint.

CASE No. 8.—Private C——, Company "I," 53d Georgia Regiment, Semmes' Brigade, McLaws' Division; health good; hair light; complexion fair; and by occupation, previous to enlistment, a farmer. Received at the battle of Sharpsburg, Maryland, September 17th, 1862, a gun-shot wound through the right elbow, fracturing both condyles. The patient refusing to submit to an operation, I removed the smaller fragments of bone and sent him across the river.

*Result.*—Recovered with complete anchylosis.

CASE No. 9.—Private S——, Company "C," 53d Georgia Regiment, Bryant's Brigade, McLaws' Division, Longstreet's Corps; of robust constitution; complexion fair; hair light; and by occupation, previous to enlistment, a farmer. Received, November 29th, 1863, in the assault on Fort Sanders, at Knoxville, Tennessee, a gun-shot wound in right arm; the ball entering three inches below the articulation of the elbow, and directly over the interosseons space—ranging upward—and becoming impacted between the two condyles. After enlarging the point of entrance, with a bistoury, I succeeded, with an elevator and ball forceps, in removing the ball. This patient was left in the hands of the enemy, in charge of my friend, Assistant Surgeon J. F. Cotton. He informed me that the patient recovered without anchylosis.

*Recapitulation.*—We have here nine cases of gun-shot wounds in elbow joint. In six, total excision was practiced; five of which we know to have been entirely successful. One—the Federal soldier—I am confident, from what I saw of him on the seventh day after the operation, made a good recovery. We then have one case in which the olecranon process alone was excised. We are satisfied, from what we saw of this case in 1865, that total excision would have been much better for the patient, and reflected more credit on the operator. The same would have been the case in No. 8. In case No. 9 the patient was exceedingly fortunate in recovering without anchylosis; though had we a similar case, we should treat it in a like manner, leaving exsection for an after consideration, as all patients have secondary excisions much better than secondary amputation. It may be asked what are the contra indications in excisions of the elbow joint? In our opinion resection should never be resorted to under the following circumstances:

1st. When we have division of the ulnar nerve.*

2d. When the principal blood vessels are injured to such an extent as to destroy their functions.

3d. When extensive injury to the soft parts has taken place.

4th. When the fracture of the humerus, radius and ulnar is so extensive as to render the arm useless, should the patient survive the operation.

Griffin, Georgia, March 20, 1866.

---

* We saw a man from Wofford's Brigade appear before the Medical Examining Board of McLaws' Division, in 1864, for retirement, upon whom the elbow had been excised. The arm was nothing but an incumbrance to him, owing to division of the ulnar nerve.

